# Characterization of Removal Reasons for Nurse Sows and the Associated Removal Due to Their Extended Lactation Length in Hyperprolific Farrow-Wean Herds

**DOI:** 10.3390/ani14111607

**Published:** 2024-05-29

**Authors:** Joab Malanda Osotsi, Peter Balogh, Gabriella Novotnine-Danko

**Affiliations:** 1 Department of Animal Science, Institute of Animal Science, Biotechnology and Natural Conservation, Faculty of Agricultural and Food Sciences and Environmental Management, University of Debrecen, Boszormenyi Street 138, 4032 Debrecen, Hungary; novotnine@agr.unideb.hu; 2 Doctoral School of Animal Science, University of Debrecen, Boszormenyi Street 138, 4032 Debrecen, Hungary; 3 Non-Independent Department of Statistics and Methodology, Institute of Statistics and Methodology, Faculty of Economics and Business, University of Debrecen, Boszormenyi Street 138, 4032 Debrecen, Hungary; balogh.peter@econ.unideb.hu; 4 HUN-REN-DE High-Tech Technologies for Sustainable Management Research Group, University of Debrecen, Boszormenyi Street 138, 4032 Debrecen, Hungary

**Keywords:** pig production, nurse sow parity, reproductive failure, heat, did not conceive

## Abstract

**Simple Summary:**

Handling of large litters in hyperprolific pig farms can only be done using a nurse sow system or artificial rearing system. Nurse sows are a group of sows used to nurse surplus piglets via extended lactation length. Understanding the husbandry aspects of the nurse sow system is required for improving pig farming practices. Furthermore, removal patterns focusing on nurse sows have not been carried out, and therefore, this research provides information that contributes to how culling decisions could be made in herds utilizing nurse sows. A high removal of young parity nurse sows affects herd structure. After weaning, nurse sows are highly removed due to failure to return to cycle and conceive. Return to estrus is considered the most important indicator of reproductive soundness of any weaned sow and therefore a crucial component in managing non-productive days in herds. Extended lactation length for nurse sows of two weeks appears to be the most appropriate period in which the nurse sow experiences a lesser chance of removal due to failure to express heat.

**Abstract:**

This study aimed to characterize and quantify reasons for the removal of nurse sows and identify the removal associated with their extended lactation length (ELL). A total of 100,756 removed nurse sows within a period of 2016–2022 from 53 sow herds in the Midwest USA were analyzed. Reproductive failure was the most common removal reason (χ^2^ = 8748.421, *p* < 0.001) affecting P1, P2, and P3 nurse sows. Failure to conceive and absence of estrus were the main causes of reproductive failure (χ^2^ = 352.480, *p* < 0.001) affecting P1 and P2 nurse sows and P1 and P5 nurse sows, respectively. When P2 and P6 nurse sows had an ELL of 0–7 d, they faced a high chance (χ^2^ = 13.312, *p =* 0.021) of removal due to conception failure and failure to return to heat, respectively. When P2 and P5 nurse sows had an ELL of 8–14 d, they were highly vulnerable (χ^2^ = 59.847, *p* < 0.001) to removal due to failure to conceive and showing heat, respectively. Finally, when ELL was at 15–21 days, P4 and P5 nurse sows were more likely (χ^2^ = 41.751, *p* < 0.001) to be removed due to failure to express heat, whereas at the same time, P2 and P3 nurse sows experienced the same removal threat due to failing to conceive. These results could help producers manage nurse sow systems.

## 1. Introduction

The removal of sows from herds provides information that contributes to how culling decisions are made. The contribution of each reason could depend on regions and management practices. Due to hypeprolificacy in modern pig production [1], management practices have led to the utilization of nurse sows. According to Baxter et al. [2], nurse sows are selected from the present lactating sows to nurse surplus piglets once they have weaned their own piglets. Nurse sows seem to portray a small proportion of the total sow herd size, ranging between 11 and 33% [3] or 10 and 15% [4]; however, cumulatively, there are many sows. A unique observation is that nurse sows are subjected to extended lactation length (ELL). Extended lactation length of nurse sows has notable negative impacts. For instance, the most profound is the loss of body reserves depicted by backfat thickness due to high milk production [5]. Continuing to nurse piglets puts pressure on the nurse sow. Therefore, sows may mobilize much of their bodily reserves, particularly in the event of insufficient lactational resources, leading to decreased backfat thickness in the first two weeks [6]. Backfat thickness indicates the amount of energy required for reproduction [7], and sows that extensively deplete these reserves show poor reproduction thereafter [8,9]. De Rensis et al. [10] found that backfat loss during lactation was negatively associated with pregnancy rate (*p* < 0.04) and positively associated with weaning-to-estrus interval (*p* < 0.01). This suggests that sows that lost a significant amount of backfat during lactation took longer to achieve subsequent estrus and had a lower pregnancy rate. According to Tantasuparuk et al. [11], ELL requires sows to produce milk continuously, which has been linked to significant body weight loss and a longer period between weaning and estrus. Similarly, Skorjanc et al. [12] found that sows with backfat losses of up to 21% during lactation had significantly longer weaning-to-estrus intervals. Furthermore, excess protein mobilization during lactation has been linked to decreased follicle maturation [13], which could result in poor reproductive performance. Longer weaning-to-estrus intervals, low pregnancy rates, and decreased follicle maturation, all associated with backfat loss during lactation, are indicators of poor reproductive performance. Poor reproductive performance is a catalyst for the immediate removal of a sow from the herd due to reproductive failure. Therefore, the objectives of this study were to (i) characterize and quantify the major reasons for the removal of nurse sows and (ii) establish the associated removal due to their extended lactation length with an emphasis on reproductive failure.

## 2. Materials and Methods

The data used in the analysis came from the Porcitec database USA 2022 belonging to a pig production company in the USA managing 53 herds.

### 2.1. General Information

Extended lactation length (ELL) was defined as the lactation of sows longer than usual, extrapolated from Oxford Dictionary 10th edition 2020. In the United States, the usual lactation length resulting in weaning of piglets has been shown to range between 18 and 25 days [14], with an average of 21 days, much earlier than the restricted 28 days in the European Union (according to European Legislation, EU Council directive 2008/120/EC). A nurse sow weans her own litter after a minimum of 21 days and weans an additional litter in the same lactation period from an unrelated sow after another 21 days [3,15]. Thus, there is a difference between extending lactation and increasing lactation. The former is a deliberate decision made by a stockperson to address the current management problem, resulting in one sow nursing two litters, whereas, in the latter, a sow maintains her litter until the end of lactation. Baxter et al. [2] categorized nurse sows into two broad groups: 1-STEP and 2-STEP. In 1-STEP, sows wean their piglets after 21 days and are given one-day-old surplus piglets to nurse, extending their lactation for another 21 days. Whereas in 2-STEP, sows wean their litter after 21 days and are given four-to-eight-day-old piglets to nurse, extending their lactation for another two weeks.

### 2.2. Herds

Herds in this study utilized conventional indoor systems. These systems are mostly intensive for raising pigs in climate-controlled facilities, with enhanced biosecurity protocols. This study was composed of farrow–wean swine operations using nurse sows to handle surplus piglets as opposed to artificial rearing. These farms employed a continuous farrowing system. This implies that the breeding of sows occurred every day, leading to daily farrowing, unlike the batch farrowing system. Farrow–wean systems are operations where pigs are raised until they are weaned between 5 and 7 kgs after 21 days and thereafter sold to wean–finish farms. A typical farrow–wean farm would have an on-site Gilt Development Unit, Gestation unit, and Farrowing unit. Nurse sows in this study were F1 Landrace × Yorkshire gilts purchased within the USA. Paternal breeds included Duroc terminal sires that produce offspring for the finishing industry. The company handles over 200,000 sows distributed among farms with an average live-born and weaned piglets of 14.63 and 12.71, respectively (company data). Cross-fostering was practiced after piglets had consumed their mother’s colostrum. Piglets were standardized by weight and moved to sows with fewer farrowed piglets within the first 24 h. Standard industry protocol indicates that as many own sows’ piglets should be left to her for nursing. For inclusion in the current study, herds had to be free from cases of Porcine Epidemic Diarrhoea (PED) and Porcine Reproductive and Respiratory Syndrome (PPRS), which are associated with low reproductive efficiency.

### 2.3. Data Description and Exclusion Criteria

The removed nurse sow data were extracted from the Porcitec database in the USA as of December 2022. The data on nurse sows were based on the fact that this study contributed to a larger project investigating the use of nurse sows in swine production. Nurse sows from the dataset were identified as those sows that had a “nurse on” event and were later removed from the herd. The dataset comprised seven years of recordings (2016–2022) from 53 herds under similar management operations. The following data were extracted from the database for each female: nurse sow tag number, parity number, reason for removal, cause of removal, and extended lactation length (in days). The choice of variables was specific, as parity number is a criterion used in selecting a nurse sow [16]. Furthermore, because the selected herds followed the company policy of culling sows beyond parity six, only nurse sows up to parity six were included in the study and referred to as P1-P6. A P1 nurse sow was a sow in parity 1 who, after weaning her own piglets, was selected to nurse surplus piglets in the same lactation. Therefore, the name “nurse sow” comes into effect after being selected. A similar understanding was used in relation to the other nurse sow parities 2–6. Gilts as parity 0 were purposefully excluded since they have not farrowed to be selected as nurse sows.

Removal reasons followed the categorization criteria of Mote et al. [17], such that the first category, “reproduction”, comprised all nurse sows removed due to six major causes of reproductive failure (RF); no heat, did not conceive, abortion, vaginal discharge, retained pigs, and failed to farrow. Removal due to RF was narrowed down to capture those nurse sows who, after weaning their second litter, failed to reach the next parity by being removed due to RF. The second category, “productivity”, comprised nurse sows removed due to farrowing and lactation-weaning productivity. The third category, “old age”, comprised nurse sows removed because of old age/parity. The fourth category, “structure”, consisted of nurse sows removed due to lameness, and finally, the fifth category, “feed intake”, contained nurse sows removed due to body condition. Three groups of ELL were created: 0–7 days, 8–14 days, and 15–21 days. This was carried out to capture the possible periods described by Baxter et al. [2] and have a variation of one week between each.

From the received dataset, nurse sows that had missing removal reasons recorded as “blank” (13,341 nurse sows, 9.4%) were excluded. These nurse sows were still active in the herds at the time of data recording, although they had a registered “nurse on” event. During the data recording, 23,979 (16.9%) nurse sows were depopulated and therefore not included in the analysis. Depopulation happened during the period of 2020, 2021 and 2022, a time of COVID-19 pandemic. The COVID-19 pandemic most likely affected the labour force in sow farms. This could have created the need to thin down herd sizes. Nurse sows that had more than 21 days as extended lactation length (3090 nurse sows, 2.1%) were excluded from the analysis. There was no repetition of nurse sow removal since once the nurse sow was culled, it exited the herd. The dataset was limited in that it lacked information about the date of birth of the sow, which could have helped in assessing her herd life. The final dataset consisted of 100,756 nurse sows.

### 2.4. Statistical Analysis

Descriptive statistics using cross-tabulations for removal reasons among nurse sow parities 1–6 were obtained by contingency tables in SPSS (statistics software version 29). The Chi-square test of independence is a statistical hypothesis test used to determine whether two categorical or nominal variables are likely to be related or not. Therefore, Chi-square tests were used to compare the frequency distributions (%) between the sow groups with different parities and removal reasons; the frequency distributions between the sow groups with different parities and the different causes of reproductive failure; the frequency distributions between the sow groups with different parities and risk of reproductive failure removal (based on “no heat” and “did not conceive” causes) among different extended lactation length. A *p*-value ≤ 0.05 was considered statistically significant. All reported *p*-values were based on a two-tailed hypothesis.

## 3. Results

### 3.1. Characterization of Major Removal Reasons

Reproductive Failure (RF) was the most common removal reason, accounting for 37.57% and affecting young parities 1, 2, and 3. Sows in P1 and P2 were at the highest vulnerability of removal due to productivity. Removal due to structure and feed intake was significant among parities 1, 2, and 3 (Table 1).

The five major reasons for removal were reproduction, productivity, old age, structure, and feed intake. Sows were removed mainly because of reproductive failure. Removal from reproductive failure increases from parity 1 to parity 3 and slows down from parity 4 to parity 6. There was an increase in the number of sows removed by productivity from parity 1 to parity 4, and a decrease was observed from parity 5 to parity 6. Many sows were removed by old age in parity 6. Removal by structure (lameness) increased from parity 1 to parity 3 and slowed from parity 4 to parity 6. Most sows removed by feed intake (body condition) increased from parities 1 to 3 and slowed down from parities 4 to 6.

### 3.2. Major Causes of Reproductive Failure Removal

Following the weaning of the second litter, the overall cumulative chance of a nurse sow being removed for failing to conceive was highest at 30.7% and affecting parity 1 and 2 nurse sows. No heat was the second cause of RF removal at 23.0% and mostly affecting parities 1 and 5 nurse sows. Parity 2 nurse sows seemed to be at a sustained threat of being removed due to other causes of RF such as retained pigs, vaginal discharge and failed to farrow. Due to the sustained likelihood of removal that P2 nurse sows faced along the way, many of them possibly never reached level P3. Similarly, P3 and P4 nurse sows were found to have a high chance of removal due to vaginal discharges. Removal due to abortion was not a threat to all parities. (Table 2).

The six main causes of reproductive failure among nurse sows were no heat, did not conceive, abortion, retained pigs, vaginal discharge, and failure to farrow. Nurse sows being removed by failure to express heat were mainly for parity 3. The tendency of removal by no heat had a downward slope from parities 3 to 6. Nurse sows failing to conceive were mainly for parity 2, and removal by failure to conceive started to slow down as parity increased with age. Removal by abortion and vaginal discharge took a pyramid shape, such that there was an increase witnessed from parity 1 to 3 and a decline from parity 4 to 6. Removal by retained pigs and failed to farrow slowed down from parity 2 to 6.

### 3.3. Reproductive Failure Removal Associated with Extended Lactation Length

The length of time a nurse sow spent in extended lactation was used to determine the associated removal. When P2 and P6 nurse sows were extended for 0–7 days, they faced a high vulnerability of removal due to failing to conceive and showing heat, respectively (χ^2^ = 13.312, *p* = 0.021). When P2 and P5 nurse sows were extended for 8–14 days, they faced a high removal due to failure to conceive and showing heat, respectively, at (χ^2^ = 59.847, *p* < 0.001). Finally, when ELL was at 15–21 days, P4 and P5 nurse sows experienced high (χ^2^ = 41.751, *p* < 0.001) removal due to no heat, whereas at the same period of time, P2 and P3 nurse sows witnessed the same threat of removal due to failing to conceive. (Table 3).

In all three durations of extended lactation length, the risk of removal by did not conceive appears to be the main cause among all parities except during 15–21 days for parity 5.

## 4. Discussion

### 4.1. Major Removal Reasons

Reproductive failure was the most common reason for removal (Table 1), in agreement with previous culling studies [17,18,19]. This removal was significant (χ^2^ = 8748.421, *p* < 0.001) among young parity nurse sows, similar to the findings in studies [20,21,22]. Removal patterns are distinct based on various contributing factors, including management protocols, parity inclusion, and even the country of research. In the USA, RF removal was indicated at 32.0% (2317/7242) by Allaire et al. [23], 33.6% (2680/7973) by Lucia et al. [24] and 35.1% (175/498) by Mote et al. [17]. These previous author’s findings are slightly lower than what is recorded in this research of 37.57% (37,852/100,752), which could be explained by the possibility of nurse sow influence. Nurse sows have been found to show decreased backfat thickness due to extended lactation [5]. This results in poor subsequent reproduction [9], thereby increasing their likelihood of removal due to RF. Nevertheless, it can be noted that there has been an increasing trend of RF removal among USA pig farms. The high removal of young parity sows destabilizes the herd structure and is associated with increased purchase of replacement gilts. A sow needs to stay in the herd for a minimum of three parities in order for the producer to realize its investment value [25,26,27], implying that such sows have the chance to recoup their initial replacement costs.

Removal due to old age was the second highest, and nurse sows in this study were recorded as removed by old age/parity. A sow can be removed due to old age by virtue of being old based on herd life days; likewise, it can also be removed by being old in relation to the current parity. This understanding could have likely informed the way of recording at the farm level, thereby triggering a new trend. A sow whose lactation is extended falls behind in days within her current lactating group, possibly interfering with her lactation interval. Nevertheless, the results of this study indicate that such removal by old age was significant (*p* < 0.001) in older parities P4, P5, and P6, in agreement with [28,29,30] studies. It should also be noted that the sows in this study were designed to be culled at P6, in line with company policy.

Productivity was the third most common reason for removal. Sows could be removed by productivity because they had fewer total-born piglets or fewer weaned piglets per lactation. In this study, young nurse sows, P1 and P2, were found to be highly vulnerable (*p* < 0.001) to being removed due to productivity, similar to the findings made by [18,30]. Furthermore, productivity removal based on lactation to weaning implies that those sows that are unable to lactate effectively and wean the “required” number of piglets are removed from the herds. Although not weaning the required number of piglets is a dynamic of many factors, sow-related factors, especially adequate mothering ability (nursing and protection), play a major role. At the farm level, it is commonly said that old sows are “experienced mothers”, perhaps due to their many lactations. However, the selection of a nurse sow has favoured young parity sows [16].

Structure in this study comprised all sows removed by lameness and was ranked fourth, highly affecting lower parities (*p* < 0.001). Studies investigating lameness as a concern in pig production have found that incidences of lameness are higher in low-parity sows than in older-parity sows [31,32,33]. In this study, there was a notable link between removed sows due to lameness and feed intake (body condition). This shows that lame sows have a challenge with adequate feeding [34,35], which affects their body condition. According to Baxter et al. [2], additional confinement in farrowing crates for nurse sows would cause welfare issues such as foot, shoulder, and leg problems, compounding their lameness situation. Feed intake, mainly implying removal due to poor body condition, was the final major removal at 4.64% (*p* < 0.001), affecting lower parities. Nurse sows are expected to continuously produce milk during ELL to meet piglet nutritional demands. However, young sows have been found to have reduced voluntary feed intake [36]. This puts them at a challenge of meeting their body growth and milk requirements for the litter, exacerbated by the fact that they are the most chosen nurse sows [16].

### 4.2. Reproductive Failure Removal Causes

Reproductive failure (RF) is an unplanned sow removal, and Boripun et al. [37] recently recommended the need to further investigate this RF removal. The study found that did not conceive was the major cause of RF removal at 30.7% (11,638/37,852). Similar findings reporting failure to conceive as the most common RF cause have been reported as follows: 37.7% (1065/2680) by Lucia et al. [24] and 37.0% (709/1913) by Koketsu et al. [38]. The per parity removal due to not conceiving was significant (χ^2^ = 352.480, *p* < 0.001) among P1 and P2 sows. Parity is an intrinsic factor affecting sow conception [39], and young sows have been found to consume less feed [40]. Less feed is inadequate for coping with their physiological demands such as growth, reproduction, maintenance, and nursing [41]. Furthermore, the well-known phenomena of Parity 2 Dip [5,42] in pig production is associated with poor reproductive performance of P2 sows. This phenomenon could be the one associated with the sustained likelihood of removal for P2 sows witnessed through vaginal discharges, failed to farrow, and retained pigs at *p* < 0.001.

No heat was the second most common cause of RF removal at 23.0% (8724/37,852). The findings in this study corroborate findings of failure to express heat as the second most common cause of RF reported by other authors as follows: 27.0% (723/2680) by Lucia et al. [24], 25.2% (482/1913) by Koketsu et al. [38] and 29.7% (244/823) by Masaka et al. [29]. No heat significantly (χ^2^ = 352.480, *p* < 0.001) affected P1 and P5 nurse sows. Among other factors, sows need adequate nutrition to express the estrus cycle; however, lower parity sows have been associated with low feed intake [36], this provides a possible chance for them failing to come to estrus. Older sows are associated with no heat due to the possible reduced ovarian activity and age-related hormonal imbalances [43,44]. Furthermore, as a sow ages, her reproductive system may become less effective, which could result in irregular or missing heat cycles [45].

In this study, removal through abortion was not significant (*p* > 0.001) among all parities, similar to the findings of Tummaruk et al. [46] when analyzing 30,058 removed sow records. Abortion may happen at any parity, although summertime heat stress has been linked to greater risks of abortion [47,48]. However, seasonality was not considered in this study. Nevertheless, when analyzing 128,535 records, Iida et al. [49] reported abortion (*p* < 0.05) in P0 and P5 sows. Gilts P0 are at higher risk of encountering pathogens in the herds as they mix for the first time with the resident sows. At the same time, an old sow’s immunity against infection could be dropping due to old age. Vaginal discharge was the third most common cause of RF, significantly (*p* < 0.001) affecting P2, P3, and P4 nurse sows. Vaginal discharges may originate from the uterus or vagina and can be observed shortly before estrus or after insemination [50]. Sows in parities 3 and 4 have farrowed at least 3 times and their uterine environment could be susceptible to infections. Furthermore, these findings could be a result of other factors, including the previously stated Parity 2 Dip influence affecting the reproductive performance of P2 sows and herd health-related issues that require attention across all parities.

### 4.3. Reproductive Failure Removal Associated with Extended Lactation Length

Based on the 1-STEP and 2-STEP approaches used in nurse sow systems [2], sow lactation can be extended for three weeks. Extended lactation length is associated with decreased backfat thickness [5], leading to poor reproductive performance [9]. Findings in this study should be understood with caution as the backfat thickness at the start and end of lactation was not measured. However, selecting P6 sows as nurse sows and extending their lactation for 0–7 days resulted in a significant (χ^2^ = 13.312, *p* = 0.021) removal for them not expressing heat after weaning, leading to their subsequent removal (Table 3). Old sows are naturally affected by ageing, resulting in less efficient reproductive tracts [45]. When a similar ELL of 0–7 days was observed in P2 nurse sows, they were significantly (χ^2^ = 13.312, *p* = 0.021) removed because they did not conceive. It is difficult to tell whether this likelihood is actually related to the ELL of P2 nurse sows or the well-known phenomena of Parity 2 Dip, which is associated with their poor reproductive performance. When the ELL was 8–14 days, it appeared that the chances of removal by no heat and failure to conceive significantly (χ^2^ = 59.847, *p* < 0.001) affected P5 and P2, respectively. When the ELL was made for 15–21 d, P4 and P5 were at high risk (χ^2^ = 41.751, *p* < 0.001) of being removed due to no heat, whereas P2 and P3 were most vulnerable (χ^2^ = 41.751, *p* < 0.001) for removal due to failure to conceive. In a study by Maes et al. [51] failure to conceive was associated with a loss of backfat during lactation, although the findings were not statistically significant (*p* > 0.05). However, there was a positive correlation between the number of weaned piglets per sow and the decrease in backfat thickness during lactation. This implied that higher numbers of weaned piglets were observed in sows that had lost more backfat. Nurse sows have been found to wean more piglets on average, 12.4 compared to 11.7 by non-nurse sows [3]. Increased dietary needs are required to match the anticipated milk production to sustain more piglets until weaning. Hillbrand and Elsaesser [52] observed that fat deposits in adipose tissues are a reservoir for the sex steroid hormone progesterone (P4), and alterations in backfat quantities affected the P4 concentration during the estrus cycle. Backfat loss during lactation would therefore likely affect the P4 levels and have an effect on the sows’ return to heat. Failure to express heat is therefore considered the first threat of reproductive failure removal that weaned sows face.

## 5. Conclusions

The results of this study have provided the reasons for the removal of sows, with a particular focus on nurse sows. Young parity nurse sows are more vulnerable to removal owing to reproductive failure and productivity. The findings of this study should be interpreted with caution because the study concentrated on the risk of reproductive failure removal resulting from extended lactation length of nurse sows.

## 6. Future Perspectives

Animal husbandry meant for human food consumption is currently being shaped by consumer preferences, which affect livestock production systems. Livestock production systems that appear to provide more natural conditions for animals are being favoured by the market as seen to be welfare-friendly. Handling large litters in intensive hyperprolific sow systems can only be performed using a nurse sow system or artificial rearing system. Nurse sow systems undoubtedly provide natural conditions for surplus piglets to suckle and experience sow–piglet interactions. Hyperprolificacy in pig production may not stop, and therefore, the nurse sow system is the only natural way to handle large litters in large-scale pig farms. Scientific findings to help improve the nurse sow system are warranted, as farmers face a future where customer satisfaction is likely to be determined by their farming practices.

## Figures and Tables

**Table 1:** Frequency (number and percentage) of nurse sows removal reasons for all parities (*n* = 100,756).

			Parity of Sow				
Removal Reason	P1	P2	P3	P4	P5	P6	Total
Reproduction	5885 (50.00%) +	7779 (48.71%) +	8174 (43.13%) +	7148 (35.68%) -	5609 (29.34%) -	3257 (21.85%) -	37,852 (37.57%)
Productivity	2028 (17.23%) +	2376 (14.88%) +	2704 (14.27%)	2818 (14.07%)	2342 (12.25%) -	1678 (11.26%) -	13,946 (13.84%)
Old age	2173 (18.46%) -	3596 (22.52%) -	5617 (29.64%) -	7917 (39.52%) +	9508 (49.73%) +	9034 (60.60%) +	37,845 (37.56%)
Structure	941 (7.99%) +	1311 (8.21%) +	1434 (7.57%) +	1294 (6.46%)	915 (4.79%) -	538 (3.61%) -	6433 (6.38%)
Feed intake	744 (6.32%) +	908 (5.69%) +	1024 (5.40%) +	858 (4.28%) -	746 (3.90%) -	400 (2.68%)	4680 (4.64%)
Total	11,771	15,970	18,953	20,035	19,120	14,907	100,756

Pearson’s Chi-square test χ^2^ = 8748.421; df = 20; *p* < 0.001; Contingency Coefficient: 0.283. Sign ‘-’ indicates value of Pearson residuals ≤ −2.0; sign ‘+’ indicates value of Pearson residuals ≥ 2.0, *p* ≤ 0.05. All percentages in columns add up to 100%.

**Table 2:** Frequency (number and percentage) of different causes of reproductive failure per parity of nurse sows (37,852).

			Parity of Sow				
Reproductive Failure Cause	P1	P2	P3	P4	P5	P6	Total
No heat	1537 (26.0%) +	1468 (18.9%) -	1703 (21.0%) -	1692 (23.7%)	1524 (27.2%) +	800 (24.2%)	8724 (23.0%)
Did not conceive	1988 (33.6%) +	2474 (31.8%) +	2444 (30.1%)	2069 (29.0%) -	1630 (29.1%) -	1033 (31.2%)	11,638 (30.7%)
Abortion	685 (11.6%)	929 (11.9%)	961 (11.8%)	818 (11.5%)	586 (10.5%)	348 (10.5%)	4327 (11.4%)
Retained pigs	425 (7.2%)	658 (8.5%) +	627 (7.7%)	532 (7.5%)	367 (6.6%)	215 (6.5%)	2824 (7.5%)
Vaginal discharge	786 (13.3%) -	1567 (20.2%) +	1734 (21.4%) +	1470 (20.6%) +	1078 (19.2%)	657 (19.8%)	7292 (19.3%)
Failed to farrow	490 (8.3%)	679 (8.7%) +	648 (8.0%)	556 (7.8%)	417 (7.4%)	257 (7.8%)	3047 (8.0%)
Total	5911	7775	8117	7137	5602	3310	37,852

Pearson’s Chi-square test χ^2^ = 352.480; df = 25; *p* < 0.001; Contingency Coefficient: 0.096. Sign ‘-’ indicates value of Pearson residuals ≤ −2.0; sign ‘+’ indicates value of Pearson residuals ≥ 2.0, *p* ≤ 0.05. All percentages in columns add up to 100%.

**Table 3:** Likelihood of removal of a nurse sow after extended lactation length based on “no heat” and “did not conceive”.

				Extended Lactation Length					
0–7 Days *				8–14 Days **			15–21 Days ***		
No Heat	Did Not	Total		No Heat	Did Not	Total	No Heat	Did Not	Total
Parity		Conceive		Conceive			Conceive		
1	271 (40.57%)	397 (59.43%)	668	630 (43.93%)	804 (56.07%)	1434	576 (44.44%)	720 (55.56%)	1296
2	252 (37.39%) -	422 (62.61%) +	674	587 (35.64%) -	1060 (64.36%) +	1647	577 (38.88%)-	907 (61.12%) +	1484
3	249 (38.19%)	403 (61.81%)	652	808 (42.68%)	1085 (57.32%)	1893	602 (40.48%)-	885 (59.52%) +	1487
4	242 (41.72%)	338 (58.28%)	580	813 (44.74%)	1004 (55.26%)	1817	585 (46.39%) +	676 (53.61%) -	1261
5	201 (43.23%)	264 (56.77%)	465	761 (48.41%) +	811 (51.59%) -	1572	517 (50.05%) +	516 (49.95%) -	1033
6	130 (48.69%) +	137 (51.31%) -	267	379 (40.75%)	551 (59.25%)	930	262 (45.25%)	317 (54.75%)	579
Total	1345 (40.68%)	1961 (59.32%)	3306	3978 (42.81%)	5315 (57.19%)	9293	3119 (43.68%)	4021 (56.32%)	7140

* Pearson’s Chi-square test χ^2^ = 13.312; df = 5; *p* = 0.021; Contingency Coefficient: 0.063. ** Pearson’s Chi-square test χ^2^ = 59.847; df = 5; *p* < 0.001; Contingency Coefficient: 0.080. *** Pearson’s Chi-square test χ^2^ = 41.751; df = 5; *p* < 0.001; Contingency Coefficient: 0.076. Sign ‘-’ indicates value of Pearson residuals ≤ −2.0; sign ‘+’ indicates value of Pearson residuals ≥ 2.0, *p* ≤ 0.05. All percentages in rows for specific extended lactation add up to 100%.

## Data Availability

None of the data was deposited in an official repository. The data that support the research findings can be obtained from the authors upon reasonable request.
